# Bridged Carbon Fabric Membrane with Boosted Performance in AC Line‐Filtering Capacitors

**DOI:** 10.1002/advs.202105072

**Published:** 2022-01-20

**Authors:** Miao Zhang, Kang Dong, Sadaf Saeedi Garakani, Atefeh Khorsand Kheirabad, Ingo Manke, Mingmao Wu, Hong Wang, Liangti Qu, Jiayin Yuan

**Affiliations:** ^1^ Department of Materials and Environmental Chemistry Stockholm University Stockholm 10691 Sweden; ^2^ Institute of Applied Materials Helmholtz‐Zentrum Berlin für Materialien and Energie Berlin 14109 Germany; ^3^ Department of Chemistry & Department of Mechanical Engineering Tsinghua University Beijing 100084 China; ^4^ Institute of Polymer Chemistry College of Chemistry Nankai University Tianjin 300071 P. R. China

**Keywords:** alternating current line filtering, phase angle, poly(ionic liquid), porous carbon membrane

## Abstract

High‐frequency responsive capacitors with lightweight, flexibility, and miniaturization are among the most vital circuit components because they can be readily incorporated into various portable devices to smooth out the ripples for circuits. Electrode materials no doubt are at the heart of such devices. Despite tremendous efforts and recent advances, the development of flexible and scalable high‐frequency responsive capacitor electrodes with superior performance remains a great challenge. Herein, a straightforward and technologically relevant method is reported to manufacture a carbon fabric membrane “glued” by nitrogen‐doped nanoporous carbons produced through a polyelectrolyte complexation‐induced phase separation strategy. The as‐obtained flexible carbon fabric bearing a unique hierarchical porous structure, and high conductivity as well as robust mechanical properties, serves as the free‐standing electrode materials of electrochemical capacitors. It delivers an ultrahigh specific areal capacitance of 2632 µF cm^−2^ at 120 Hz with an excellent alternating current line filtering performance, fairly higher than the state‐of‐the‐art commercial ones. Together, this system offers the potential electrode material to be scaled up for AC line‐filtering capacitors at industrial levels.

## Graphical abstract

A polyelectrolyte complexation‐induced phase separation strategy is developed to produce hierarchically porous carbon fabric membrane with nanoporous carbon linker as the flexible electrode materials of ultrafast electrochemical capacitors (ECs). Corresponding device is capable of delivering an ultrahigh *C_A_* of 2632 µF cm^−2^ at 120 Hz with an excellent AC line filtering performance, much higher than the state‐of‐the‐art commercial AECs.

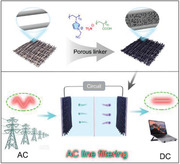

## 1 Introduction

Flexible ultrafast electrochemical capacitors (ECs) with efficient alternating current (AC) line‐filtering functions play a key role in the development of flexible electronics for the internet of things (IoT) and portable devices.^[^ ^1^, ^2^, ^3^, ^4^, ^5^ ^]^ Commercial aluminum electrolyte capacitors with bulky and rigid attributes, which have dominated the market for a long history, however are facing challenges to meet the ever‐increasing demand of these devices requiring miniaturized, lightweight and flexible circuits.^[^ ^6^, ^7^, ^8^ ^]^ Thus, searching for electrode materials for ECs that deliver integrated hierarchical microstructure, high conductivity, and mechanical flexibility represents one of the most active areas of current research of advanced energy materials.^[^ ^9^, ^10^, ^11^, ^12^, ^13^ ^]^

Among various electrode materials that enable ECs with decent AC line‐filtering functions, of particular interest are the porous carbon materials due to their low cost, large surface areas, metal‐free nature, and chemical stability, as well as tunable conductivity and electrochemical activity.^[^ ^7^ ^]^ Regrettably, so far powdery porous carbon materials‐based ECs for line‐filtering application suffered from low charge/discharge rate because of significant interfacial resistance across the boundary of carbon particulates, especially tortuous pores in powdery carbons force them to store charges in a distributed fashion, undermining the rate capability of corresponding devices when driven by 120 Hz AC. In addition, gluing these porous carbon particulates with electronically insulating polymer binders into membrane‐like electrodes is common but inevitably deteriorates the conductivity, porosity, and long‐term operational stability.^[^ ^14^ ^]^ Binder‐free assemblies of carbon nanomaterials, e.g. graphene and carbon nanotube, featured good conductivity yet somehow moderate performance because of the difficulty in controlling of the desired pore structure.^[^ ^15^, ^16^ ^]^ Further advances in this rising field pose a challenging task for materials design and synthesis to achieve a simultaneous optimization of highly conductive, exquisite pore structure and mechanical robustness with little‐to‐no trade‐offs.

In this context, efforts have been devoted to developing nanoporous carbon membrane electrodes for ECs with AC line‐filtering functions. Commercial carbon fiber‐based woven and non‐woven porous membranes with a combined profile of excellent mechanical strength, outstanding electrical conductivity, good flexibility, and lightweight take a striking position in electrochemistry‐related fields.^[^ ^17^, ^18^ ^]^ However, owing to their low specific surface area, they are not fully suitable as active electrode materials to be used in ECs, which require a large ion‐accessible surface. Recently, explosive interests have been directed to create porous and/or heteroatom‐doped carbon fibers, and then convert them into predominant active components for energy storage or electrocatalysis.^[^ ^19^ ^]^ Creating pores on carbon fibers will amplify their surface area and make them directly applicable in energy storage devices. Most of the methodologies developed so far have been derived from the following three traditional concepts: I) post‐treated carbon fibers by using corrosive chemical agents (e.g., H_3_PO_4_ and KOH) or gas (e.g., CO_2_ and H_2_O) to create micropores,^[^ ^20^ ^]^ ii) utilizing soft or hard template by controlling fiber precursors,^[^ ^21^ ^]^ and iii) tailoring parameters of fabrication techniques, such as electrospinning, to engineer pores during fiber formation before carbonization. These methods are generally time‐ and/or cost‐intensive and will compromise the conductivity and mechanical properties of the carbon paper.

Herein, we proposed a fundamentally disparate strategy to produce porous linkers in carbon fabric membrane, which can satisfy all the requirements of electrode materials for ultrafast ECs. Poly (ionic liquid), a group of ionic liquid‐derived polyelectrolytes, was judiciously chosen as a porogen as well as a carbon precursor to fill in and link the carbon fabric membrane via novel inter‐polyelectrolyte complexation methods developed by us. Upon carbonization process, it generated a robust, binder‐free porous fabric membrane directly serving as electrodes for ECs. In this design, the carbon fabric skeleton with high conductivity and mechanical strength was preserved and further equipped with porous linkers which intimately bridged interconnected carbon fibers. The as‐fabricated ECs delivered an ultrafast rate performance at 120‐Hz with a high specific areal capacitance of 2632 µF cm^−2^, which is significantly higher than the benchmark AECs and prevails the graphene‐based materials.

## 2 Results and Discussion

### 2.1 Preparation and Structure Control of Porous Filler‐based Carbon Membrane

**Figure 1:**
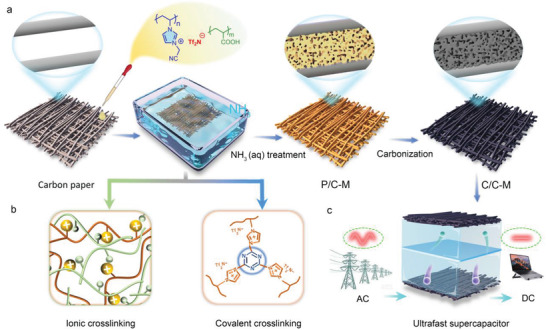
a) Schematic illustration for the fabrication process of poly(ionic liquid)‐enabled porous carbon fabric membrane. The poly(ionic liquid) used here is PILTf_2_N. b) Scheme for the dual crosslinking mechanism: i) ionic crosslinking from polyelectrolyte complexation and ii)covalent crosslinking from cyclization reaction are illustrated in yellow square. c) Schematic showing AC line filtering application of the C/C‐M derived supercapacitor.

Cationic poly(ionic liquid), poly(3‐cyanomethyl‐1‐vinylimidazolium bis(trifuoromethane sulfonyl)imide) (abbreviated as PILTf_2_N, where Tf_2_N denotes the counter anion) and poly(acrylic acid) (abbreviated as PAA) were chosen jointly as the filler precursor with dual‐functions: i) high‐yield carbon source with satisfactory conductivity of resultant N‐doped carbon; ii) pore generating agent via the Tf_2_N‐based molecular templating mechanism.^[^ ^22^, ^23^ ^]^ The detailed synthesis and structural characterization of PILTf_2_N were provided in supporting information (Figure S1, Supporting Information). The fabrication was performed via a scalable bottom‐up method (**Figure** **1**). In brief, PILTf_2_N as a strong polycation and PAA as an anionic weak polyelectrolyte were first dissolved in dimethyl sulphoxide (DMSO) in a molar ratio of 1:1 for the imidazolium cation to the –COOH unit. The PAA was protonated in DMSO, a nonprotic solvent, and stayed neutral in charge, so at this stage, PILTf_2_N and PAA were mixed homogeneously in solution without electrostatic complexation. This was the prerequisite for following solution‐based processing. Then, a defined amount of a transparent polymer blend solution was cast onto a pristine carbon fabric paper and dried at 80 °C for one hour. The polymer/carbon composite membrane was immersed into an aqueous ammonia solution, which is a necessary step to create macropores in the polymer domains that are hosted by the composite membrane. These beneficial macropores will be later brought to the carbon products as important ion transport highways and electrolyte reservoirs. Once the cast and dried membrane is in contact with aqueous (aq.) NH_3_ solution, the NH_3_ and water molecules will penetrate into the composite membrane simultaneously. Three physicochemical processes are initiated side by side: i) Ammonia molecules will deprotonate the –COOH group of PAA into –COO^−^NH_4_^+^ ion pair of the anionic poly(ammonium acrylate), which driven by entropy will ionically crosslink with cationic PILTf_2_N near to form interpolyelectrolyte complex (Figure 1e). ii) The diffusion of water as a poor solvent into PILTf_2_N will cause phase separation of the hydrophobic PILTf_2_N bearing Tf_2_N^−^ to form pores. In parallel, the pores are stabilized by the robust interpolyelectrolyte complex during drying. iii) The weak basic environment arising from ammonia triggers covalent crosslinking of the nitrile group into a triazine‐based network. This novel catalytic cyclization reaction of the distal nitriles in PILTf_2_N at room temperature was recently discovered by us,^[^ ^24^, ^25^ ^]^ and the successful implementation of this reaction in the current system was confirmed by the Fourier transform infrared spectroscopy (FTIR) characterization (Figure S2, Supporting Information). IR bands located at 1659 cm^−1^ emerge with respect to the untreated material, which corresponds to the *ν*C═N IR bands in the *s*‐triazine rings, indicating the occurrence of covalent crosslinking.^[^ ^24^ ^]^ Collectively, the facile aq. NH_3_ treatment directed the delicate porous structure under a complicated multiple ionic and covalent crosslinking mechanism. In this case, aq. NH_3_ plays triple roles for the pore formation and stabilization, that are deprotonation agents, phase separation solvent as well as catalyst for covalent crosslinking. With this porous membrane in hand, the free‐standing porous carbon membrane with integrated structure was achieved via pyrolysis and was then employed directly as an electrode for EC devices in a two‐electrode configuration using 3 M H_2_SO_4_ as a liquid electrolyte. For the sake of clarity, the polymer/carbon composite membrane was abbreviated as **P**/C‐M, and its carbonized one as C/C‐M. Four kinds of mass loadings of polymer blend were employed with increasing polymer content from 0.2, 0.4, 0.6, to 1.3 mg cm^−1^, which are abbreviated as C/C‐M_1_, C/C‐M_2_, C/C‐M_3,_ and C/C‐M_4_, respectively.

### 2.2 Structural Characterization of the Hierarchical Porous Microstructure

**Figure 2:**
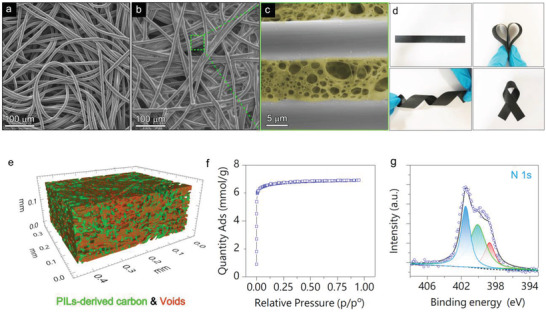
SEM images of a) pristine carbon fabric membrane. b) PILTf_2_N/PAA glued carbon fabric membrane. c) enlarged view from (b). d) Photos show the flexibility of C/C‐M. e) 3D rendering image of C/C‐M. (Red color: PILTf_2_N/PAA‐derived carbon. Green color: voids). f) N_2_ adsorption–desorption isotherms of carbonized PILTf_2_N/PAA porous membrane. g) XPS spectrum of the high resolution of N_1s_ of pristine carbonized PILTf_2_N/PAA porous membrane.

The unique features of the P/C‐M and C/C‐M designed here lie in their interconnecting binary components (the carbon fibers and the in situ generated N‐doped carbon glue) coupled with a hierarchical porous structure with two size levels. The fabric carbon membrane consists of microfibers (about 10 µm in diameter) that act as conductive and mechanical support to provide primary pores (type I pores) of tens to hundred µm in size (**Figure** **2**a). Such large pores will help keep the mechanical integrity of the membrane during bending or twisting as a flexible device. After drop‐coating the polymer blend solution, drying at 80 °C, and soaking in an aq. ammonia solution, a hierarchical porous structure was detected by scanning electron microscopy (SEM) characterization (Figure 2b,c). Interestingly, the carbon microfibers were connected by a polymer matrix with discrete submicron‐to‐micron pores (type II pores), and most of the porous polymer was positioned in the interspace of the fabric instead of their surface as confirmed by EDS elemental mappings (Figure S3, Supporting Information). After carbonization, the secondary pores of smaller size are well preserved, as verified by the similar morphology in the same region (Figure S4, Supporting Information). Such maintenance of conformal filler/linker morphology even after a high‐temperature carbonization process at 900 °C arises from the strong adhesion nature of polymer blends to the carbon fibers and a high carbonization yield of PILTf_2_N at high temperature. Even though these filling materials may affect the flexibility of the carbon membranes to some extent due to the reduction in large porosity, the C/C‐M still demonstrates excellent mechanical flexibility, which can be folded to arbitrary shapes at will (Figure 2d). More impressively, the membrane exhibits nearly constant resistance even after 100 bending/unbending cycles (Figure S5, Supporting Information). The morphology of the porous linking part retains integration as well (Figure S5, Supporting Information). This is of key importance for flexible and portable devices for special fields because most of the flexible devices require a power supply to transfer AC to DC or the circuit included in the devices requires flexible filtering units. The judicious choices of polymers and solvents and treatment by the aq. NH_3_ solution are all crucial for the pore formation and the hierarchical pore microstructure.^[^ ^26^ ^]^ In comparison, the coated membrane without aq. NH_3_ solution treatment presented a nonporous structure by SEM characterization in the polymer region between neighboring carbon fibers. (Figure S6, Supporting Information). Both the carbon fiber and the polymer in the case without aq. NH_3_ pre‐treatment showed a smooth surface with a compact structure that is inappropriate for the target supercapacitor application. A pristine porous polymer membrane without carbon paper as substrate was also prepared as a reference. After aq. NH_3_ treatment, the surface of this membrane presents an exquisite porous structure (Figure S7, Supporting Information). Thickly dotted pores were spotted in the SEM Image. Even though a similar porous structure was obtained without a fabric substrate, the pure polymer‐derived carbon membrane in a freestanding form was too brittles to be used in flexible devices, which limits their practical value. Here, the fabric carbon paper as a substrate was essential from a viewpoint of mechanical properties.

Synchrotron X‐ray tomography was employed for three‐dimensional visualization and quantification of the internal pore structure and pore distribution. As shown in the 3D rendering of the composite membrane, the discrete pores in SEM images appeared to be evenly distributed throughout the entire membrane in an interconnected manner, which is desirable for fast mass transfer during the charging/discharging process in the electrochemical test (Figure 2e, Figure S8a, Supporting Information). 3D volume analysis after data reconstruction revealed that the void fraction in a 3D sub‐volume is about 16.57%, and that most of the voids have a volume smaller than 50 µm^3^ (Figure S8b, Supporting Information). These interconnected macrospores are a necessity when considering high‐frequency operation that requires rapid diffusion of ions and electrons in an electrochemical capacitor device. Meanwhile, they can function as reservoirs for the liquid electrolyte. The surface area of the pristine PILTf_2_N/PAA derived porous carbon membrane was investigated and calculated by using the Brunauer–Emmett–Teller (BET) equation. The nitrogen adsorption increased sharply at low pressures (P/P_0_ < 0.05, Figure 2f) due to the nitrogen filling in micropores (<2 nm), A high S*_BET_* value of 602 m^2^ g^−1^ was obtained, which was attributed to the template effects of the counteranion Tf_2_N^−^.^[^ ^22^ ^]^ Microspores derived from carbonization and decomposition of the polymer matrix, and macrospores existing already in the hybrids due to phase separation of the polymer blends, together formed the unique hierarchical porous structure in the C/C‐M. Moreover, as an additional benefit of N‐rich PILTf_2_N as a carbon precursor, effective nitrogen dopant was introduced into the carbon matrix. High resolution of N1s XPS spectrum indicates that the N mainly existed in the form of graphitic N (35%), pyridinic N (20%), and pyrrolic N (45%) (Figure 2g). Some of these doped N atoms will better the wettability of the electrode surface to the electrolyte.^[^ ^27^ ^]^ As shown in Figure S7, Supporting Information, the N‐doped C/C‐M presented favorable wettability in a 3 M H_2_SO_4_ electrolyte and precipitated down to the bottom of the vial. By contrast, the pristine carbon fabric membrane without N doping only floated on the surface of the electrolyte, indicative of poor affinity to the electrolyte. (Figure S9, Supporting Information) Noted that, N doping in carbon materials has been proven to hold positive effects in AC line filtering application in our previous studies due to better affinity to the electrolyte.^[^ ^28^ ^]^

### 2.3 Electrochemical Performance for Symmetric Supercapacitor

The porous fabric membrane with unique porous glue/linkers is a suitable candidate as an electrode material for ultrafast electrochemical capacitors. Conventional carbon materials with tortuous pores in a powdery form as electrode materials are far from satisfactory in terms of areal capacitance density at 120 Hz (C*_A_* ^120^), absolute phase angle value at 120 Hz (*ϕ*_120_), and characteristic frequency (*f_0_*). For a high‐frequency electrochemical capacitor electrode material, high conductivity, and straightforward or extensive pore‐based structure are mandatory requirements for adequate frequency response at 120 Hz.^[^ ^7^ ^]^ Bearing these in mind, we consider the ideal electrode materials should have a robust network free of insulating binder to ensure high conductivity and open/large pores to facilitate mass transfer and circumvent distributed charge storage.^[^ ^29^ ^]^ The porous filler‐based membrane prepared here with a binary carbonaceous heterostructure can meet all these demands. First, the mechanically robust carbon fabric paper (2.59 MPa in tensile strength tests) with a high graphitization degree and large primary pores gives rise to a highly conductive network, which ensures a low electrode resistance (Figure S10, Supporting Information). Second, the PILTf_2_N‐derived porous filler brings open, interconnected, and “electrolyte‐friendly” secondary pores, thus improving the ion‐accessible surface area of carbon fabric substrate. Generally, there is a trade‐off between being more porous and having a higher conductivity for carbon membranes. Herein, PILTf_2_N‐derived porous filler with a relatively lower graphitization degree served as a linker intimately adhered onto the highly graphitized carbon fibers. This bridge‐like structure counteracted the negative effect of the comparably lower conductivity of PILTf_2_N–derived carbon. Meanwhile, this intimate interfacial connection resulted in much reduced interfacial resistance. It was demonstrated that reducing the contact resistance between the current collector and the electrode material as well as within the active material is critical for achieving a high‐frequency response.^[^ ^36^ ^]^ Raman spectrum characterization was carried out to identify the structural heterogeneity of the two kinds of carbon materials in the membrane. Three peaks located at 1350, 1588, and 2702 cm^−1^ are derived from the D, G, and 2D bands of carbon materials, representing a breathing mode of *sp^2^* carbon rings, planar configuration *sp^2^* bonded carbon, and the second‐order of the D band (or an overtone of the D band) (Figure S11, Supporting Information), respectively. The decreased *I_G_/I_D_* value for carbon fibers from 1.05 to 0.91 with respect to that of porous filler/linker as well as a more pronounced 2D peak for pristine carbon fibers reflect a higher graphitization degree for the former.

**Figure 3:**
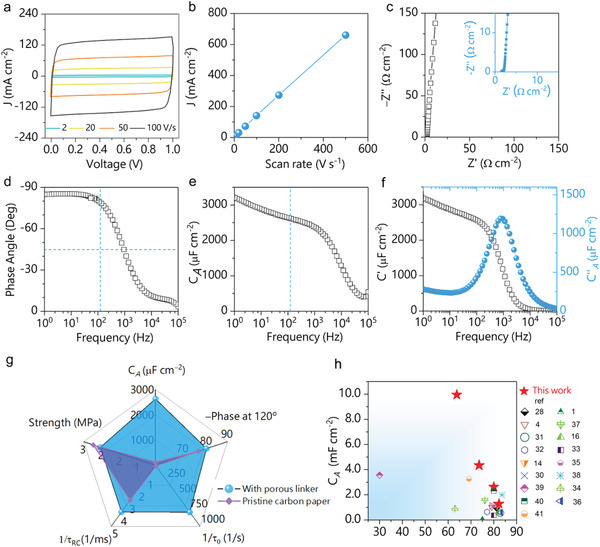
Electrochemical performance of the C/C‐M_1_‐based EC. a) Cyclic voltammetry curves at different scan rates. b) Evolution of discharging current density versus scan rate. c) Nyquist plot of impedance (inset, an enlarged view in high‐frequency region) d) Plot of impedance phase angle versus frequency. e) Plot of areal capacitance (*C_A_*) versus frequency using a series‐RC circuit model. f) Plot of the real and imaginary part (C′ or C”” of specific capacitance versus frequency. g) Radar plot of pristine carbon fabric and porous glued carbon fabric for performance comparison. h) Ashby diagram of the phase angle versus specific areal capacitance for the C/C‐M electrodes compared with reported film‐based electrodes. ^[^ ^1^, ^4^, ^14^, ^16^, ^28^, ^30^, ^31^, ^32^, ^33^, ^34^, ^35^, ^36^, ^37^, ^38^, ^39^, ^40^, ^41^ ^]^

With all the above prominent merits integrated within a single binary porous carbon membrane, the corresponding symmetric EC cells were constructed by employing the C/C‐M directly as electrodes to simulate actual device behaviors (3 M sulfuric acid aqueous solution was chosen due to its high ion conductivity ≈ 0.8 S cm^−1^). The loading of the porous polymer fillers was finely controlled by the volume and concentration of the casting polymer solution. Here, membranes with an areal mass loading of 1.33 mg cm^−2^ (C/C‐M_1_) for polymer were used for a primary sample to characterize their electrochemical performance. Their performance was first evaluated by cyclic voltammetry (CV) measurements at different scan rates. All profiles at various scan rates exhibit quasi‐rectangular profiles, indicating fast ion/electron transport and minimal distributed charge storage throughout the electrode (**Figure** **3**a). For an ideal capacitor capable of response at high frequency, the current density response should be linearly proportional to the scan rate according to the definition of capacitance (C = *I* scan rate^−1^). In this case, a linear dependence of discharge current density on scan rates up to 500 V s^−1^ was achieved, suggesting excellent capacitive behavior (Figure 3b). Besides, a galvanostatic charge‐discharge test (GCD) was carried out to further evaluate its rate capability at various current densities. All profiles were in a symmetric triangular shape, without any noticeable internal resistance drop (IR drop), even at a fast discharge rate of less than 0.2 s, as presented in Figure S12, Supporting Information. The very low equivalent series resistance (ESR) in the cell indicates superior conductivity and good contact within the electrode materials. To further reveal their frequency response characters at 120 Hz, electrochemical impedance spectroscopy (EIS) measurement was carried out, and the derived data are plotted in Figure 3 c–f. In the Nyquist plots, all the spectra exhibited nearly vertical lines and were free of an obvious semi‐circle feature at the high‐frequency region, indicative of a capacitive nature at high frequency. An extremely low ESR of 0.05 Ω cm^−2^ was read out, further confirming the excellent conductivity of the electrodes. Impedance phase angle at 120 Hz was generally accepted as a “Figure of merit” to evaluate the line‐filtering potential for domestic electronic circuits. The closer the phase angle approaches −90°, the more capacitive behaviors the device performs. Also, there is a trade‐off between specific areal capacitance and impedance phase angle. In this study, C*_A_* ^120^ of 2632 µF cm^−2^, 1283 µF cm^−2^, 4345 µF cm^−2^, and 9946 µF cm^−2^ coupled with phase angles at −80^o^, −82^o^ and 73.6 ^o^, −65 ^o^ were achieved for C/C‐M_1_, C/C‐M_2_, C/C‐M_3_, and C/C‐M_4_ respectively (Figure 3 d,f, Figure S13, Supporting Information). To the best of our knowledge, these values are among the best records ever reported for carbon‐based materials and far higher than the commercial AECs if not considering voltage window (Figure S14, Supporting Information). Moreover, an extremely long cyclic stability was tested by GCD at 8 mA cm^−2^. After 50 000 cycle tests, there is no detectable capacitance degradation, reflecting excellent structural and chemical stability of the electrode material (Figure S15, Supporting Information). Such high C*_A_* ^120^ combined with an adequate phase angle at 120 Hz can be ascribed to the hierarchical porous structure, which provides more ion‐accessible surface area.

In a control experiment, it was found that the bare carbon membrane electrode ends up with inferior C*_A_* ^120^ compared to that of C/C‐M_1_ (Figure S16, Supporting Information). It is noteworthy here that the porous linker only accounts for 6% mass of the carbon fabric substrate, while the C*_A_* ^120^ of the final membrane increased by nearly 20 times. A radar plot was employed to demonstrate the benefit of introducing polymer linkers into bare carbon membranes (Figure 3g). For all the performance metrics, including *C_A_*, phase angle at 120 Hz, *τ*_0_, and *τ*_RC_, C/C‐M_1_ are much better than those of bare carbon fabric substrate. Impressively, considering *C_A_*, the phase angle at 120 Hz, the carbon membranes presented in this work are much better than previously reported film‐shaped electrodes, such as porous carbons. graphene and carbon nanotube (Figure 3h).^[^ ^1^, ^4^, ^14^, ^16^, ^28^, ^30^, ^31^, ^32^, ^33^, ^34^, ^35^, ^36^, ^37^, ^38^, ^39^, ^40^, ^41^ ^]^

Given various application contexts of line‐filtering capacitors, high operation voltages are frequently needed. Unlike AECs, which can improve the voltage by increasing dielectric layer thickness, the voltage windows of supercapacitors are primarily limited by their electrolyte. Here, to counterplay this issue, we take two strategies to extend the voltage window of C/C‐M: i) connecting cells in series, and II) constructing hybrid electrochemical capacitor devices with conducting polymer using 1 M NaSO_4_ as electrolyte. For the former, two cells were connected in series, the CV profile retained a rectangular shape, and the voltage window was extended to 2 V without compromising its frequency response performance (Figure S17, Supporting Information). For the latter, porous PEDOT:PSS was employed as the positive electrode, and C/C‐M_2_ served as the negative electrode (Figure S18, Supporting Information). An optimized voltage window of 0–1.8 V was achieved after matching the loading of active materials in both electrodes. From the impedance data, this hybrid device demonstrated decent frequency response, which was superior to graphene‐based hybrid electrochemical capacitors.^[^ ^15^ ^]^

### 2.4 Proof of Concept for Practical ac Line Filtering Application

**Figure 4:**
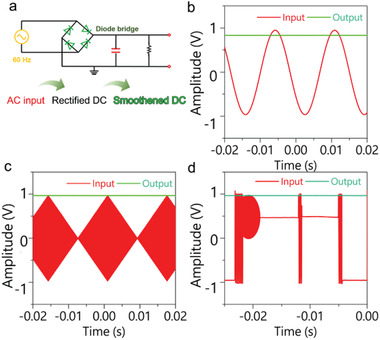
a) Schematics demonstrating the filtering circuit equipped with rectifier and EC. b) Alternating current (AC) line filtering performance of the circuit for 60 Hz sinusoidal AC input. c,d) The filtering performances of the circuit for arbitrary waveforms. The input signals are (c) rhombus and letter PIL.

As a proof of concept for real domestic line‐filtering application, a power supply line filtering circuit equipped with a C/C‐M‐based EC was constructed as illustrated in **Figure** **4**a. In this circuit, a wave generator supplies a 60 Hz AC sine wave, which will pass a full‐bridge rectifier circuit and be converted to a 120 Hz pulsating DC signal. Then the C/C‐M‐based ECs integrated into the circuit served as a current reservoir to smooth out ripples, resulting in stabilized DC output. The device with higher *C_A_* will result in smaller ripples and a lower output dc level than the counterpart with the lower *C_A_* (Figure S19, Supporting Information). Besides sinusoidal AC input signal, this circuit is capable of filtering arbitrary waves, indicating its vast potential for diverse filtering requirements in various scenarios (Figure 4 b–d).

## 3 Conclusion

In summary, we established a fabrication strategy towards fiber‐based porous carbon membranes with a brand new binary porous microstructure concept. By leveraging poly(ionic liquid)‐based polyelectrolyte complex chemistry, a porous filler was produced via a facile casting‐and‐soaking process. Carbon fabric membrane with high conductivity and large inner voids was filled and linked by this porous polyelectrolyte complex. Combined with conformal carbonization, the as‐derived porous carbon fabric membrane with hierarchical porous structure was successfully fabricated and used for ultrafast electrochemical capacitor for AC line filtering application. The corresponding electrode delivers a C*_A_* of up to a record‐high 2632 µF cm^−2^ with an impedance phase angle of 80^o^, surpassing the state‐of‐the‐art commercial ones.

## Conflict of Interest

The authors declare no conflict of interest.

## Supporting information

Supporting Information

## Data Availability

Research data are not shared.
