# Supplementary material for: Bridged Carbon Fabric Membrane with Boosted Performance in AC Line‐Filtering Capacitors
Source: Adv Sci (Weinh). 2022 Jan 20;9(7):2105072. doi: 10.1002/advs.202105072 (PMC8895147; doi:10.1002/advs.202105072)
Supplement: Supplementary file 1 — Supporting Information [file ADVS-9-2105072-s001.pdf]

WILEY-VCH

ADVANCED SCIENCE
Open Access

Supporting Information

for *Adv. Sci.*, DOI: 10.1002/advs.202105072

Bridged Carbon Fabric Membrane with Boosted Performance in AC Line-Filtering Capacitors

Miao Zhang, Kang Dong, Sadaf Saeedi Garakani, Atefeh Khorsand Kheirabad, Ingo Manke, Mingmao Wu, Hong Wang , Liangti Qu*, Jiayin Yuan**

Supporting Information

Bridged Carbon Fabric Membrane with Boosted Performance in AC Line-Filtering Capacitors

Miao Zhang[a] *, Kang Dong*[b]*, Sadaf Saeedi Garakani*[a]*, Atefeh Khorsand Kheirabad*[a]*, Ingo Manke*[b]*, Mingmao Wu*[c]*, Hong Wang** [d]*, Liangti Qu*[c]**, Jiayin Yuan**[a]

DOI: 10.1002/anie.2021XXXXX

[a]Dr. M. Zhang, Prof. J. Yuan
Department of Materials and Environmental Chemistry, Stockholm University, 10691 Stockholm, Sweden.
E-mail: jiayin.yuan@mmk.su.se

[b]Dr. K Dong, Dr. I. Manke
Institute of Applied Materials, Helmholtz-Zentrum Berlin für Materialien and Energie, 14109 Berlin, Germany

[c]Dr. M. Wu, Prof. L. Qu
Department of Chemistry, & Department of Mechanical Engineering, Tsinghua University, Beijing, 100084, China
E-mail: lqu@mail.tsinghua.edu.cn

[d]Prof. H. Wang
Institute of Polymer Chemistry, College of Chemistry, Nankai University, Tianjin, 300071, P. R. China
E-mail: hongwang1104@nankai.edu.cn

Experimental Section

Chemicals

1-Vinyl imidazole was purchased from Alfa Aesar Sweden. 2'-Azobis(isobutyronitrile) (AIBN) was purchased from Fuchen Chemical Reagents Co., Ltd., Tianjin, China. Lithium bis(trifluoromethane sulfonyl)imide (LiTFSI, 99.95%) was purchased from Io-li-tec. Poly(acrylic acid) (PAA) (Mw – 25 * 10^4 Da) was purchased from Fujifilm Wako Chemicals. Hydrochloric acid (HCl, VWR, 37 %), *aq.* NH_3 solution (Sigma-Aldrich), sodium sulfate (VWR), sulfuric acid (H_2SO_4, VWR, 95%), poly(3,4-ethylenedioxythiophene), and polystyrene sulfonate (PEDOT:PSS, Heraeus, CleviosTM PH 1000) were directly used as received from the suppliers without further purification. Carbon paper (Freudenberg H14C9) was washed with ethanol and deionized water before use. Graphite foil (conductivity~16*10^3 S.cm$^-$1, thickness = 16 μm, Emitac New Material Technology Co. Ltd, Suzhou, China) was cleaned with ethanol before use. Cellulose membrane as sacrificing template of PEDOT:PSS was purchased from NKK. Cellulose filter paper for separator was purchased from VWR. All solvents were of analytical grade.

Synthesis of cyanomethyl-3-vinylimidazolium bromide (CMVImBr) monomer, poly(1-cyanomethyl3-vinylimidazolium bromide) (PCMVImBr) and poly(1-cyanomethyl-3-vinylimidazoluim bis(trifluoromethane sulfonyl)imide) (PCMVImTf$_2$N)

Briefly, 1-vinyl imidazole (20 g, 0.212 mol) and bromoacetonitrile (30 g, 0.252mol) were dissolved in 200 mL diethyl ether and added into a round bottom flask. The mixture was stirred at room temperature overnight, then filtered off, and the solids were washed for three times with diethyl ether. The solid white powder of CMVImBr was dried under vacuum at 50 ^oC overnight. Then, CMVImBr (40 g, 0.188 mol), AIBN (308 mg, 1.88 mmol), and DMSO (400 mL) were added into a 1000 mL schlenk flask equipped with a magnetic stirrer. The solution was deoxygenated three times by a freeze-pump-thaw procedure, refilled with nitrogen. Then the polymerization was conducted at 80 ^oC for 12 h, cooled down to room temperature, and the reaction mixture was precipitated in THF, filtered off, and washed three times to thoroughly remove residual monomers. The PCMVImBr was dried in vacuum at 60 ^oC for 12 h.

PCMVImTf$_2$N (further abbreviated as PILTf$_2$N in the main text) was prepared through anion exchange between PCMVImBr and bis(trifluoromethane sulfonyl)imide lithium salt. An excess of lithium bis(trifluoromethane sulfonyl)imide aqueous solution was added dropwise into aqueous PCMVImBr solution under stirring. The PCMVImTf$_2$N precipitates were obtained, washed with deionized water,

dried in vacuum at 70 °C for 24 h. Proton nuclear magnetic resonance (^{1}H-NMR) spectrum of $PCMVImTf_2N$ was shown in Figure S1.

.

Fabrication of electrodes and symmetric electrochemical capacitor (ECs)

0.2 g of $PCMVImTf_2N$ and 0.036 g of PAA (of a defined molecular weight as mentioned above) in terms of 1:1 molar ratio of imidazolium/carboxylate were dissolved homogeneously in 2 ml of DMSO. Electrodes for ECs were prepared by drop-casting the $PCMVImTf_2N$/PAA blend onto a finely shaped carbon paper (0.25 cm^2). After drying at 80 ℃ for 1 h., the coated carbon paper was soaked into 0.2 wt% aqueous ammonia solution. The resultant carbon papers were thoroughly washed with deionized water then dried at room temperature. For the carbonization process, coated carbon papers were heated to 300 °C at a heating rate of 2 °C min^{-1} and held at 300 °C for 1 h, then followed by heating to 900 °C at a heating rate of 2 °C min^{-1} and held at 900 °C for 1 h. After that, the samples were cooled down to room temperature and directly used as electrodes to construct symmetric devices without further modification. The typical symmetric ECs were fabricated by sandwiching a porous cellulose paper as a separator between two identical electrodes. Two pieces of platinum foil were used as current collectors; clamps of the workstation were directly connected to the platinum foil. An aqueous H_2SO_4 solution (3 M) was dropped onto electrodes as a liquid electrolyte.

Fabrication of electrodes and hybrid electrochemical capacitor (HECs)

Preparation of positive electrode: A cellulose membrane (NKK) was deposited on a precleaned graphite foil. Deionized water was employed to assist the adhesion of the cellulose and graphite foil. The wet cellulose membrane was partially dried by a spin-coating machine. Then PH1000 (PEDOT:PSS) solution was dropped on the semi-dried cellulose membrane followed by a spin-coating treatment to ensure uniform coating of the PEDOT:PSS onto the cellulose microfiber. Afterward, the wet membrane was immediately soaked into a concentrated sulfuric acid solution for more than 24 h at room temperature. During this process, the cellulose membrane was gradually degraded by the acid, leaving the porous PEDOT:PSS film on the graphite foil. The as-prepared PEDOT:PSS coated graphite foil was cut into a square shape. These tailored electrodes were then cleaned with dilute sulfuric acid (12 M, 6M, and 3M) and deionized water step by step. Finally, the as-prepared electrodes were stored in diluted sulfuric acid solution (3 M) for further use.

Carbonized membrane from polymer blend drop-casting and pyrolysis were employed as negative electrodes.

Hybrid electrochemical capacitor (HEC) was fabricated by assembling positive and negative electrodes with $NaSO_4$ as a liquid electrolyte in a sandwiched manner.

Synchrotron X-ray tomography

Synchrotron X-ray tomography was conducted at the BAMline station at BESSY II (Helmholtz-Zentrum Berlin für Materialien und Energie, Germany). The X-ray beam was monochromatized to 16 keV using a double multilayer monochromator system with an energy resolution (ΔE/E) of about 3%. The detector system comprised of a 60-μm thick $CdWO_4$ scintillator, a 10x magnification lens, and a PCO4000 camera with a 4008 × 2672 pixel CCD sensor. The detector system was placed at a 15 mm distance to the sample in order to work in the in-line phase contrast mode. To increase the signal-to-noise ratio, a camera binning (2 x 2) was applied, generating images with a pixel size of around 0.88 μm with a field of view of 1.7 x 1.2 mm^2. Each dataset contains 2256 projections over 180° and 170 flatfields. An exposure time of 1.0 s was used for each projection/flatfield.

The obtained raw projections were normalized exploiting flatfields using Python code available at the beamline followed by 3D reconstruction using codes programmed in IDL 8.2. After reconstruction, pixel classification and image segmentation were performed with the ilastik software[1]. The following quantification analysis of the acquired 3D datasets was conducted using the Fiji software. Three-dimensional image rendering was performed using VGSTUDIO MAX 3.1.

Electrochemical measurements

Electrochemical measurements were performed on BioLogic potentiostat. Symmetrical two-electrode cell configurations were employed to simulate the actual device behavior. Three electrode system was used to probe the stable voltage window of single electrode. Cyclic voltammograms (CV), galvanostatic charge-discharge (GCD) tests, and electrochemical impedance spectroscopy (EIS) were carried out to characterize device performance. Cycling stability was tested by galvanostatic charge-discharge for 50,000 cycles at a charge/discharge current density of 8 mA cm^{-2}. For the AC-line filtering test, all the input signals were supplied by a 33511B arbitrary function generator (Agilent Technologies Inc., Tektronix, USA), and the signals were performed with a GBPC3005W ready-made single-phase silicon bridge rectifier (Sep Electron. Corp., Taibei, China). All outputs were recorded by the use of a RTB2002 mixed domain oscilloscope (Rohde & Schwarz, Germany).

The specific areal capacitance of electrodes, C_A ($\mu F\ cm^{-2}$), was calculated from EIS spectra by eq 1:

$$C_A = -2 / s(2\pi f Z'') \qquad \text{eq 1}$$

where f is the frequency (*Hz*), Z' is the imaginary resistance (Ω) and s is the area of the electrode (cm^2).

The RC time constants (τ_{RC}), the time required for charging to 63.2% of the full potential of the capacitor, were calculated using the eq 2:

$$\tau_{RC} = -Z' / 2\pi f Z'' \qquad \text{eq 2}$$

The real (C') and imaginary (C'') parts of electrodes were calculated using the following eq 3 and eq 4:

$$C' = -2Z'' / (2\pi f |Z|^2 s) \quad \text{eq 3}$$

$$C'' = 2Z' / (2\pi f |Z|^2 s) \quad \text{eq 4}$$

where $|Z|$ is the absolute value of the impedance (Ω), f is the frequency (Hz), Z' or Z'' is the real or imaginary components of impedance, s is the geometrical area of the electrode.
The relaxation time constant (τ_0) can be derived from the frequency (f_0) at which C'' reaches the maximum value ($\tau_0 = 1/f_0$, which means the minimum time required for discharging a capacitor with an energy efficiency > 50%).

Characterizations

^{1}H-NMR spectra were recorded at room temperature using a Bruker DPX-400 spectrometer operating at 400 MHz. DMSO-d_6 was used as a solvent for the measurement. Scanning electron microscopy (SEM) and energy-dispersive spectroscopy (EDS) elemental analysis were carried out on a JEOL JSM-7000F (JEOL, Japan) microscope with an accelerating voltage of 10 kV and 3 kV. Samples were coated with a thin gold layer for 60 seconds before the examination. The Raman spectra were recorded on a LabRAM HR800 Raman spectrometer. Fourier transform infrared (FTIR) spectroscopy was conducted on IR spectrometer (670-IR). X-ray photoelectron spectroscopy (XPS) analysis was carried out by Thermo Escalab 250XI under ultra-high vacuum conditions in the range of ~ 10^{-10} mbar using a monochromatic Al Kα X-ray source (hυ = 1486.6 eV) operated at 150 W. Binding energies were referenced to the C1s peak of (C-C, 284.8 eV) bond and the deconvolution of the core-level spectra was carried out using XPSPEAK software. Background contributions were subtracted using a Shirley function. Mechanical tensile tests were conducted on an Instron 5960 universal testing machine (Instron, USA) with a constant loading rate of 10% strain per minute with a gauge length of 15 mm.

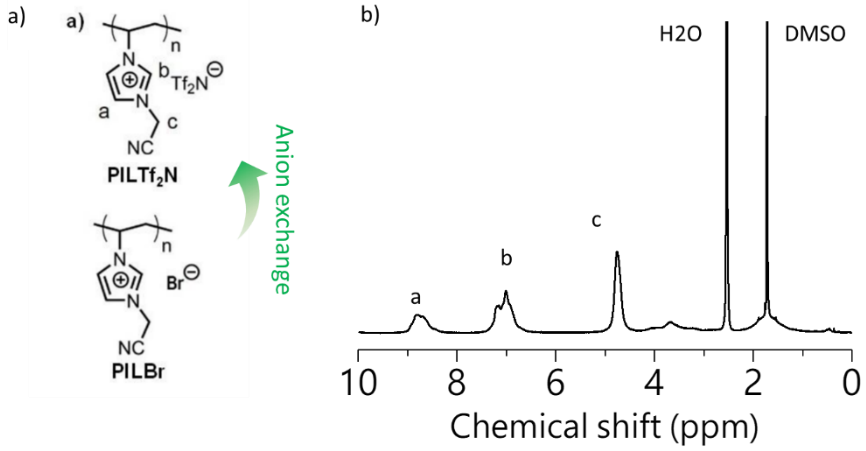

Figure S1. (a) Chemical structures of PILTf$_2$N and PILBr; (b)^{1}H-NMR spectrum of PILTf$_2$N in DMSO-d$_6$**.**

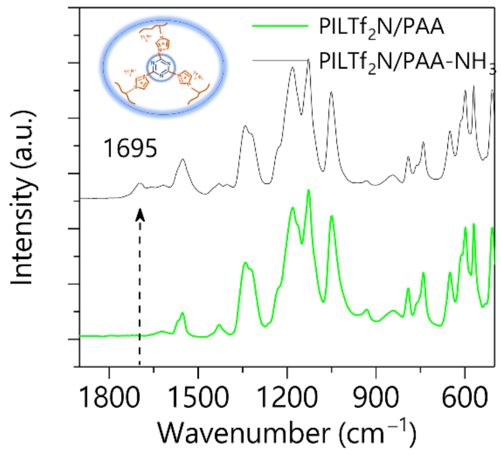

Figure S2. FTIR spectra of PILTf$_2$N/PAA composite with (black) and without (green) aq. NH_3 solution treatment.

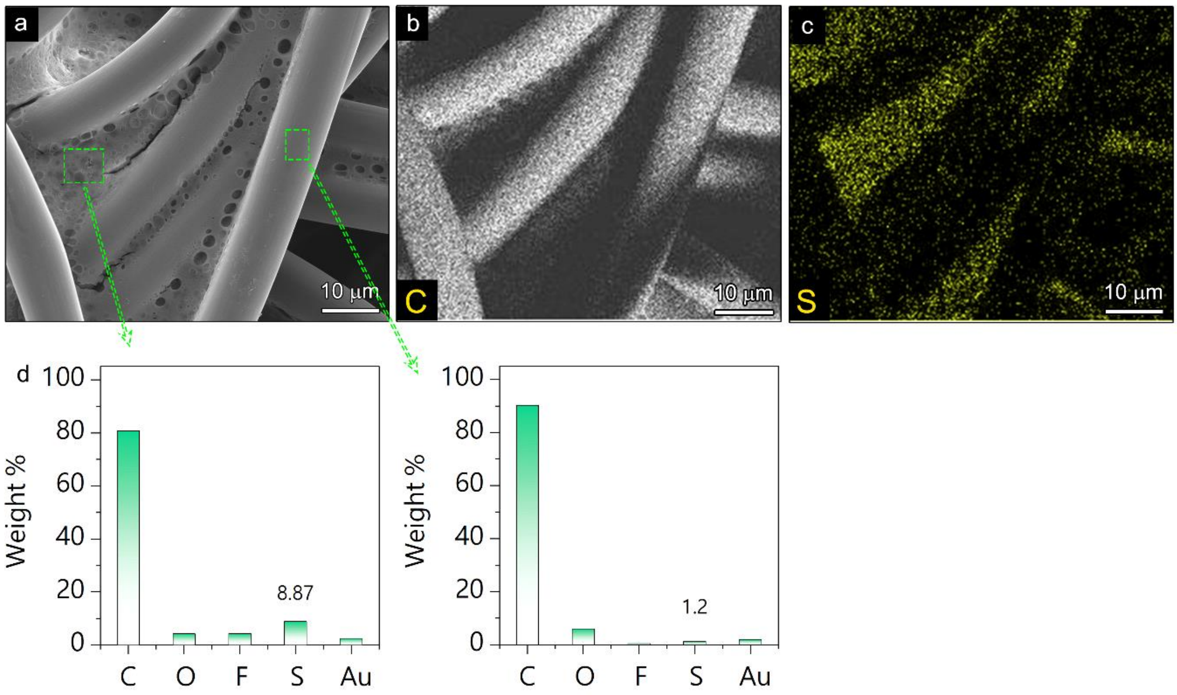

Figure S3. (a) SEM images of the PILTf$_2$N/PAA glued carbon membrane (P/C-M) and (b, c) corresponding EDS elemental mapping of C and S elements. (d) EDS analysis of the membrane before carbonization: C, O, F, S, and Au element content in the porous polymer glue and carbon microfiber. Au comes from the thin Au layer sputtered onto the sample surface before the measurement.

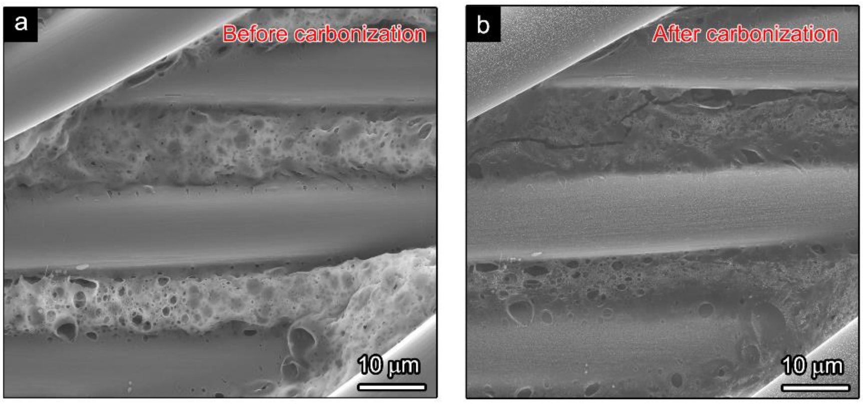

Figure S4. SEM images of the PILTf$_2$N/PAA glued carbon membrane (P/C-M) (before (a) and after (b) carbonization.

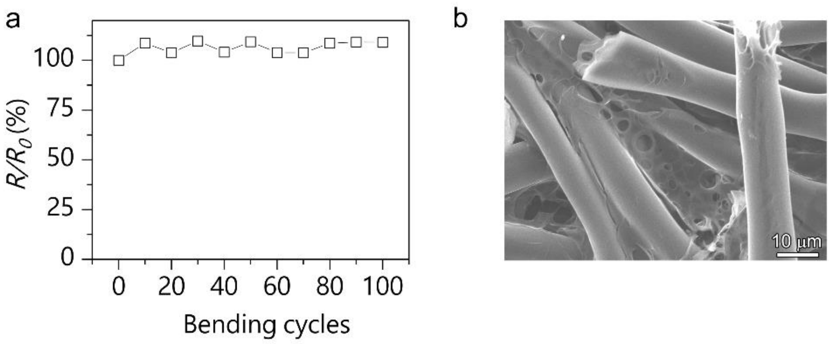

Figure S5, Bending stability of the C/C-M electrode. (a) Relative resistance (R/R_0) variation during the cyclic bending-unbending test. R/R_0 is the ratio of electrode resistance under the bending state to the initial one under the flat form. (b) SEM image after bending test.

Figure S6. SEM images of the $PILTf_2N$/PAA coated carbon membrane without ammonia solution soaking treatment.

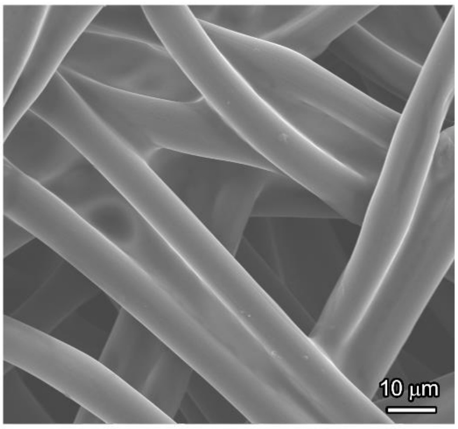

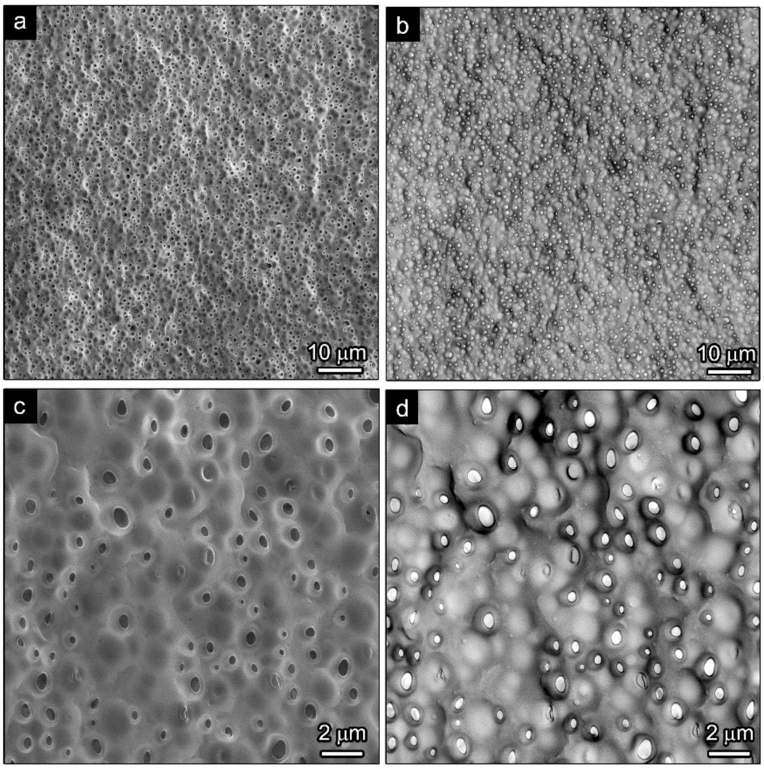

Figure S7. SEM images show the morphology of the pristine PILTf$_2$N/PAA porous membrane without carbon fabric membrane as a substrate. (a) SEM image of the membrane surface, (b) Inverse phase image of (a). (c) Enlarged view of (a). (d) inverse phase image of (c). Note: inverse phase images here are used for clearly showing the inner pores.

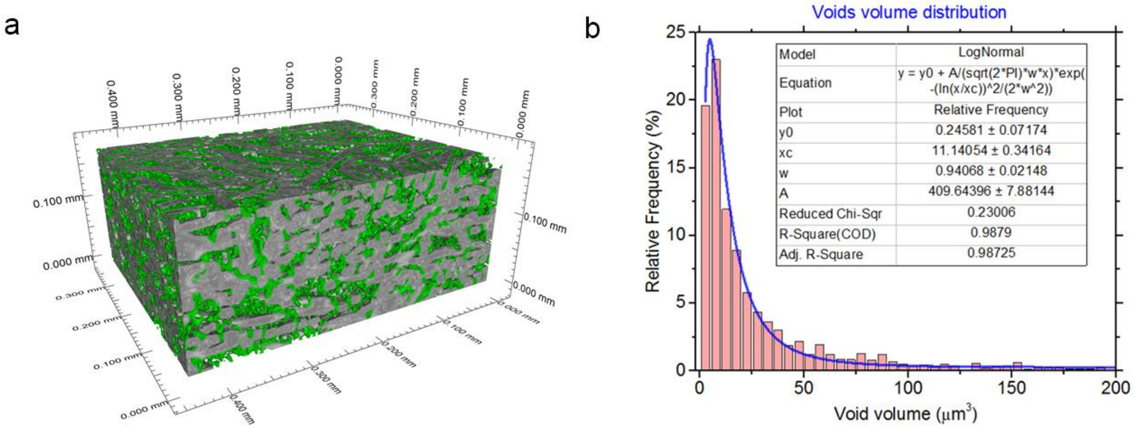

Figure S8. (a) 3D rendering image of the porous carbon membrane with carbon glue. (Grey color: carbon fiber (Green color: voids). (b) Void volume distribution obtained from synchrotron X-ray tomography.

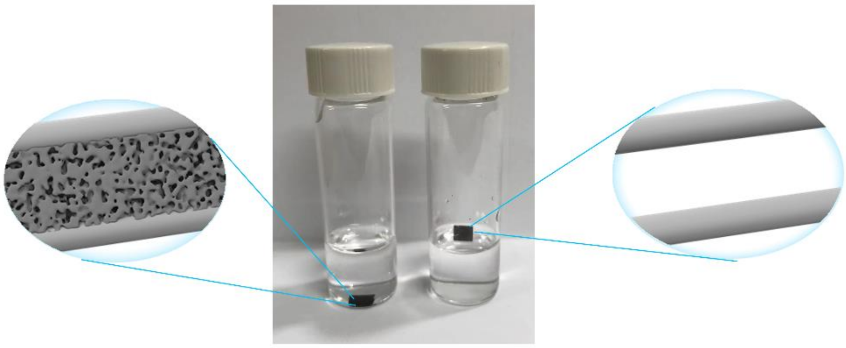

Figure S9. Wettability comparison of the porous carbon membrane with PILTf$_2$N/PAA linker. and pristine one in 3 M H_2SO_4 electrolyte.

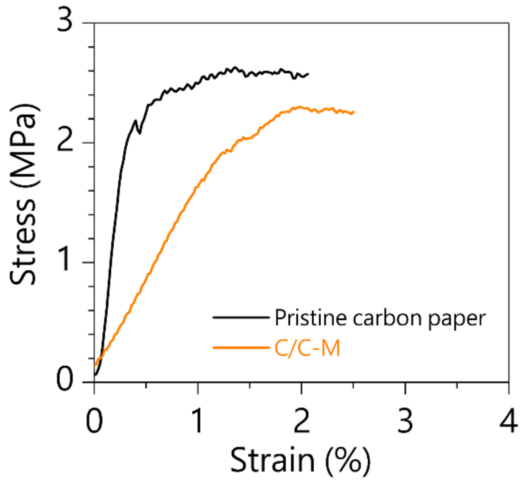

Figure S10. Tensile stress-strain curves of the pristine carbon membrane and a porous carbon membrane with carbonized PILTf$_2$N/PAA linker (C/C-M).

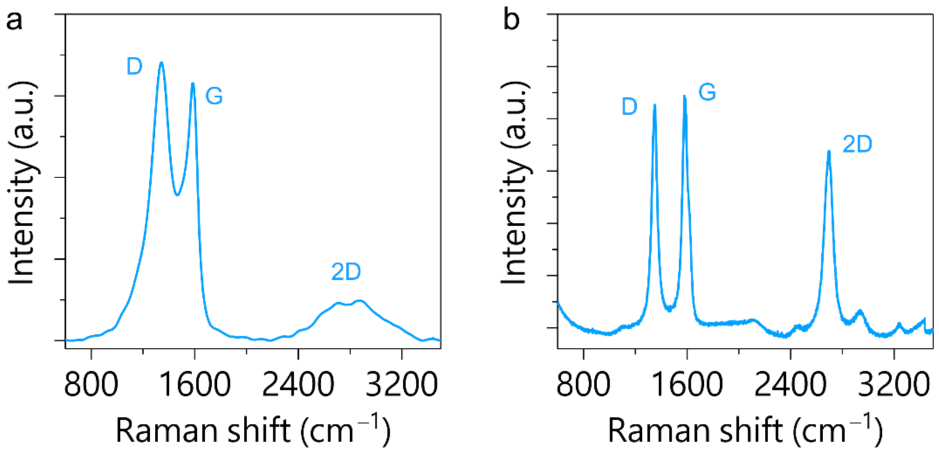

Figure S11. Raman spectra of (a) the carbonized PILTf$_2$N/PAA and (b) the pristine carbon paper.

.**Figure S12**. Galvanostatic charge-discharge curves of the C/C-M$_1$ at various current densities.

Figure S13. Electrochemical performance of C/C-M$_2$, C/C-M$_3$, C/C-M$_4$. (a) Plots of impedance phase angle versus frequency for various samples. (b) Plots of areal capacitance versus frequency using a series-RC circuit model.

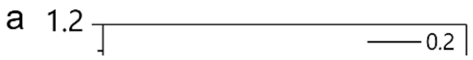

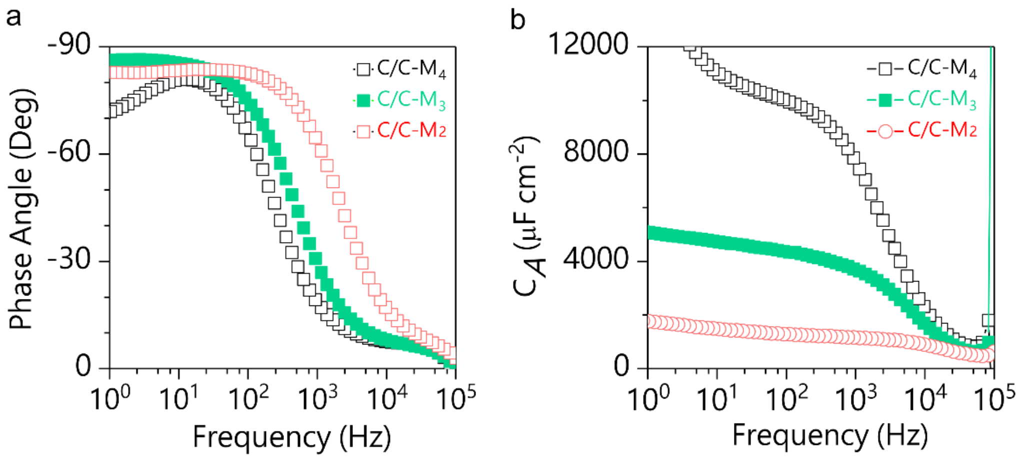

Figure S14. Performance comparison of C/C-M_1 versus commercial AEC.

Figure S15. Cycling stability test of C/C-M_1 for 50, 000 charge-discharge cycles.

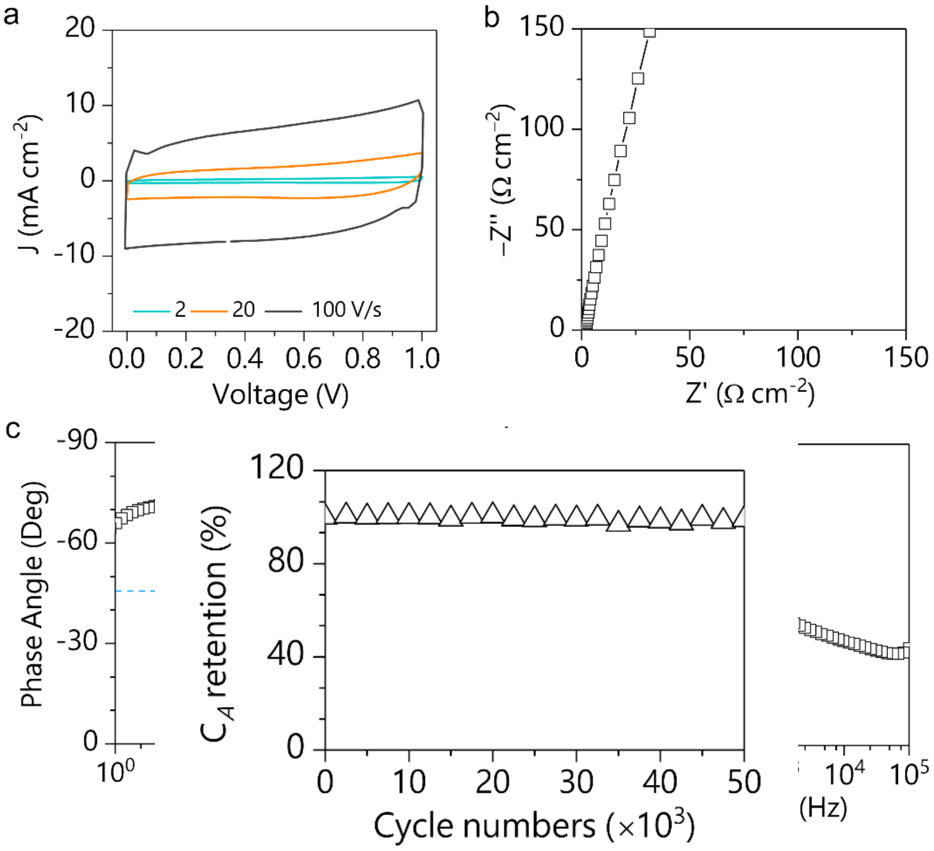

Figure S16. Electrochemical performance of the pristine carbon membrane. (a) CV curves at various scan rates. (b) Nyquist plot. (c) Plots of phase angle as a function of frequency. (d) Plots of areal specific capacitance (C_A) *versus* frequency.

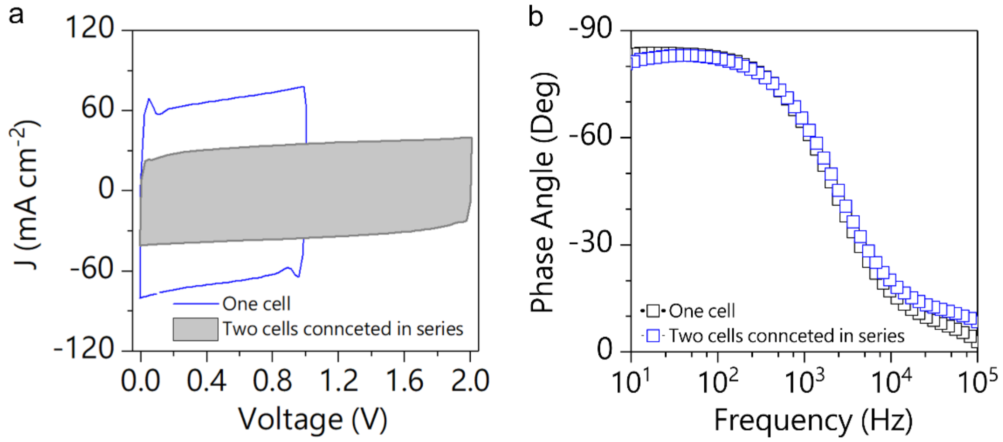

Figure S17. Electrochemical performance of two cells connected in series. (a) CV curves at a scan rate of 100 V/s. (b) Plots of impedance phase angle versus frequency of different numbers of ECs connected in series.

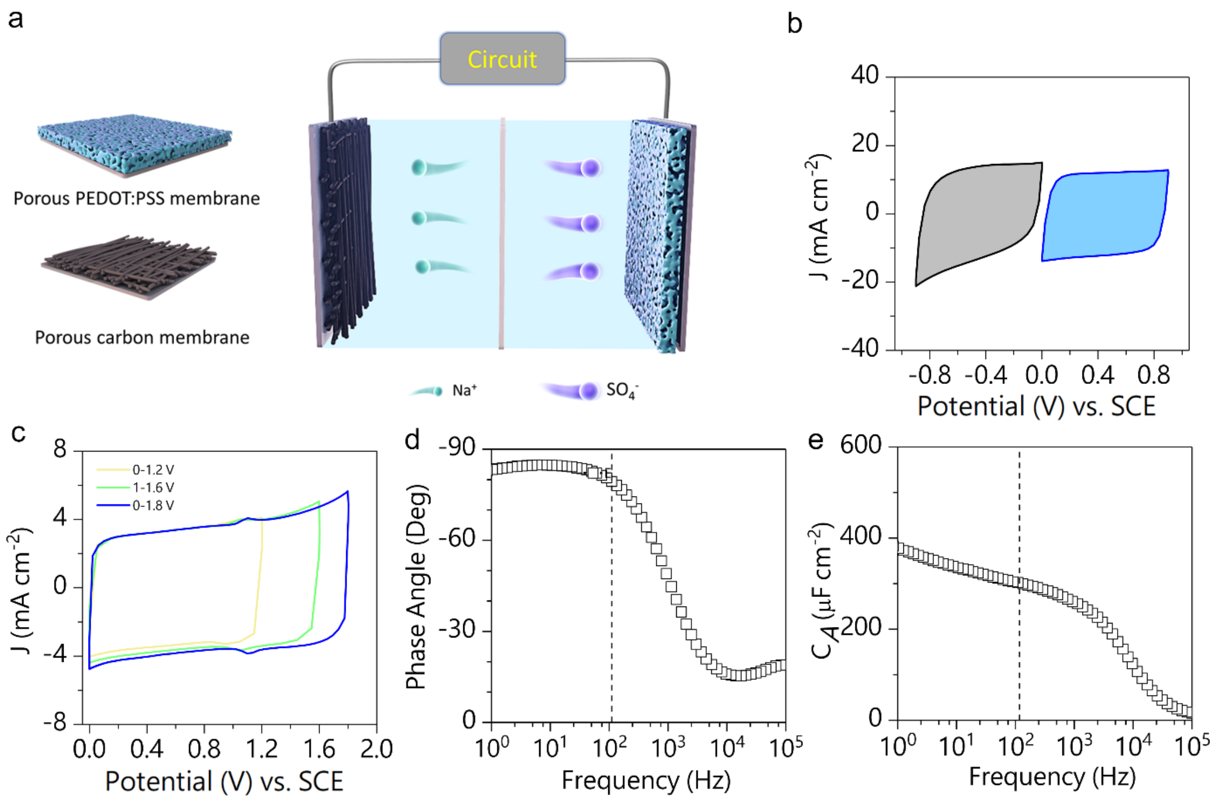

Figure S18. Electrochemical performance of the aqueous hybrid electrochemical capacitor devices (HEC). (a) Schematic illustrations of the hybrid device using 1 M $NaSO_4$ as the electrolyte. (b) CV curves of PEDOT:PSS and C/C-M_1 electrodes in separate potential windows at a scan rate of 10 V s^{-1}. (c) CV curves of the aqueous hybrid electrochemical capacitors in different voltage range at scan rate of 10 V s^{-1}. (d) Plots of phase angle versus frequency. (e) Plots of areal specific capacitance (C_A) as a

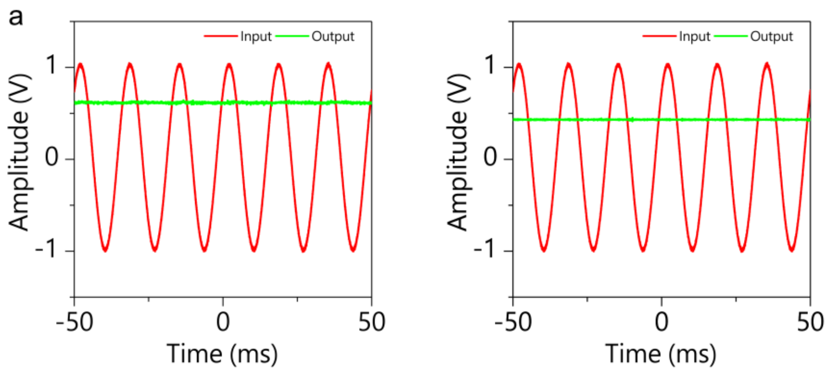

function of frequency.

Figure S19. AC filtering performance of the filtering circuits coupled with two supercapacitors of different capacitances (a) 20 μF cm^{-2}, (b) 2206 μF cm^{-2}. A loaded resistance (1000000 Ω) is incorporated in parallel.
